# The Role of Phospholipase C Signaling in Macrophage-Mediated Inflammatory Response

**DOI:** 10.1155/2018/5201759

**Published:** 2018-02-08

**Authors:** Liqian Zhu, Clinton Jones, Gaiping Zhang

**Affiliations:** ^1^College of Veterinary Medicine and Jiangsu Co-innovation Center for Prevention and Control of Important Animal Infectious Diseases and Zoonoses, Yangzhou University, 48 Wenhui East Road, Yangzhou, Jiangsu 225009, China; ^2^Department of Veterinary Pathobiology, Center for Veterinary Health Sciences, Oklahoma State University, Stillwater, OK 74078, USA; ^3^College of Animal Science and Veterinary Medicine, Henan Agricultural University, Zhengzhou, Henan 450002, China

## Abstract

Macrophages are crucial members of the mononuclear phagocyte system essential to protect the host from invading pathogens and are central to the inflammatory response with their ability to acquire specialized phenotypes of inflammatory (M1) and anti-inflammatory (M2) and to produce a pool of inflammatory mediators. Equipped with a broad range of receptors, such as Toll-like receptor 4 (TLR4), CD14, and Fc gamma receptors (Fc*γ*Rs), macrophages can efficiently recognize and phagocytize invading pathogens and secrete cytokines by triggering various secondary signaling pathways. Phospholipase C (PLC) is a family of enzymes that hydrolyze phospholipids, the most significant of which is phosphatidylinositol 4,5-bisphosphate [PI(4,5)P2]. Cleavage at the internal phosphate ester generates two second messengers, inositol 1,4,5-trisphosphate (IP3) and diacylglycerol (DAG), both of which mediate in diverse cellular functions including the inflammatory response. Recent studies have shown that some PLC isoforms are involved in multiple stages in TLR4-, CD14-, and Fc*γ*Rs-mediated activation of nuclear factor kappa B (NF-*κ*B), mitogen-activated protein kinase (MAPK), and interferon regulatory factors (IRFs), all of which are associated with the regulation of the inflammatory response. Therefore, secondary signaling by PLC is implicated in the pathogenesis of numerous inflammatory diseases. This review provides an overview of our current knowledge on how PLC signaling regulates the macrophage-mediated inflammatory response.

## 1. Introduction

Inflammation is part of the complex biological response of body tissues to harmful stimuli, such as pathogens, damaged cells, or to molecular “irritants,” and is a protective response involving both cellular and molecular mediators [1, 2]. Initially, both pro and anti-inflammatory signals with opposing effects are tightly regulated in a balanced status [3]. However, a disruption of this balance can result in an excessive inflammatory response resulting in cellular and tissue damage [4–6]. From extensive study, it has long been recognized that macrophages play a critical role in the initiation, maintenance, and resolution of inflammation.

Together with dendritic cells (DCs) and monocytes, macrophages are major components of the mononuclear phagocyte system. Macrophages participate in all phases of the immune and inflammatory responses [7]. Unstimulated macrophages are typically quiescent; however, stimulation of these cells by local micromilieu signals, however, results in their acquiring a polarized phenotype [8] either proinflammatory M1 macrophages or anti-inflammatory M2 macrophages. M1 macrophages, generally induced by LPS and IFN*γ*, generate high levels of proinflammatory cytokines [e.g., interleukin 1*β* (IL-1β), interleukin 6 (IL-6), interleukin 12 (IL-12), and tumor necrosis factor (TNF-*α*)] and oxidative metabolites [e.g., nitric oxide (NO) and ROS]; M2 macrophages stimulated by a variety of stimuli (e.g., IL-4/IL-13 and glucocorticoids) are important in the resolution of inflammation [9, 10]. Macrophages express a repertoire of pattern recognition receptors (PRRs) such as Toll-like receptors (TLRs), CD14, nucleotide-binding oligomerization domain-like (Nod-like) receptors, and RIG-I-like receptors [11–15]. This sensor array enables them to recognize a diverse range of ligands and to initiate quickly appropriate responses, such as phagocytosis, and immunomodulation through production of various cytokines [3, 14, 16]. Macrophages have elaborate strategies for the regulation of the inflammatory response.

Stimuli, such as lipopolysaccharide (LPS) and cytokines, activate macrophages by ligation of corresponding receptors, such as Toll-like receptors (TLRs) [14]. Upon activation, a variety of intracellular signals are triggered to promote the production of proinflammation cytokines [e.g., IL-1*β*, IL-6, and TNF-*α*], chemokine [e.g., macrophage inflammatory factor (MIP-1*α*) and IL-8], and toxic molecules (e.g., NO and ROS) [17, 18]. The “cytokine storm” characterized by the hyperinduction of proinflammatory cytokines and chemokines is a pathogenic mechanism resulting in some pathogens causing tissue injury and multiorgan dysfunction [19–21]. For example, the lethal lung inflammation due to infection by influenza virus (e.g., 1918 H1N1 and H5N1) and porcine reproductive and respiratory syndrome virus (PRRSV) is mainly caused by cytokine storms induced by these viral infections [20, 22–24]. Macrophages are the major source of proinflammatory mediators [25–27] and are therefore implicated in the pathogenesis of numerous inflammatory diseases.

Members of the phospholipase C (PLC) family are thus involved in intracellular and intercellular signal transduction. Accumulated evidence has demonstrated that the PLC signaling inhibitor U73122 attenuates both acute and chronic inflammation mediated by macrophages both in vivo and in vitro [28–30], linking PLC signaling to macrophage-mediated inflammation. The involvement of PLC*β*, *γ*, and *δ* in macrophage-mediated inflammation has been extensively studied, and herein the corresponding mechanisms are summarized and discussed.

## 2. The Spectrum of Expression of PLC Isoenzymes in Macrophages

PLC family enzymes are activated by numerous factors such as neurotransmitters, growth factors, histamine, and hormones, as reviewed by Nakamura and Fukami [31]. PI(4,5)P2 is the preferred substrate of PLC. Hydrolysis of PI(4,5)P2 leads to the generation of IP3 into the cytoplasm and DAG in the membrane. IP3 triggers the release of Ca^2+^ from intracellular stores, and DAG mediates the activation of protein kinase C (PKC). The activation of PKC and calcium signaling in turn activate downstream signaling [31, 32]. Concomitantly, PI(4,5)P2 also directly regulates a variety of cellular functions, including phagocytosis [33].

**Figure 1:**
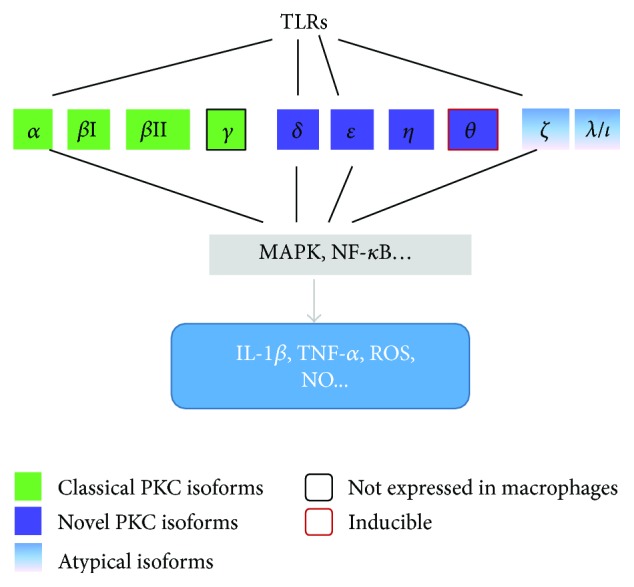
The expression of PKC isoforms in macrophages and their role in TLR-mediated inflammatory response. Among them eight, PKC isoforms (PKC*α*, *β*I, *β*II, *δ*, *ε*, *η*, *ζ*, and *λ*) are expressed in macrophages. PKC*α*, *δ*, *ε*, and *ζ* are directly related to TLR-induced inflammatory response. PKC*θ* expression in macrophages cannot be detected, but its expression can be induced by LPS/IFN*γ* stimulation.

Protein kinase C (PKC) is a family of protein serine/threonine kinases that are involved in the phosphorylation of serine and threonine amino acid residues on other proteins, or other members of this family [34]. The PKC isoforms are divided into 3 subfamilies based on their activation requirements: classical PKCs (calcium dependent) (PKC*α*, *β*I, βII, and *γ*), novel PKCs (calcium independent) (PKC*δ*, *ε*, *η*, and *θ*), and atypical PKCs (PKC-*ζ* and *λ*/*ι*) [35, 36]. According to the literature, eight PKC isoforms (PKC*α*, *β*I, *β*II, *δ*, *ε*, *η*, *ζ*, and *λ*) are expressed in macrophages [37]. Though macrophages do not express detectable PKC*θ*, its expression is upregulated in response to LPS/IFN*γ* stimulation [38], suggesting that PKC*θ* expression in macrophages is inducible by certain inflammatory stimuli. It has been known that PKC inhibitors reduce LPS-stimulated cytokine secretion by macrophages, linking PKC activation to TLR4 signaling. It has been further evidenced that PKC*α*, *δ*, *ε*, and *ζ* are directly involved in multiple steps in TLR4 pathways, as well as in the downstream activation of inflammation pertinent signaling, such as MAPK and NF-*κ*B [36, 39, 40]. PKC*θ* and PKC*ε* also activate NF-*κ*B-dependent pathways in muscle cells to promote expression of proinflammatory cytokines and chemokine [41]. PKC*ε* regulates NF-*κ*B-mediated NO production by macrophages in response to LPS stimulation [42]. Classical PKCs are critical components that control IRF-3-dependent gene expression downstream of TLR3 and TLR4 [43]. The role of PKC isoforms in TLR-dependent signaling transduction has been summarized in Figure 1. In view of the diversity of the PKC family and that PKC signaling is regulated by PLC enzymes, this further emphasizes the importance of PLC signaling in macrophage-mediated inflammation.

Currently, there are a total of 6 classes of PLC isoenzymes discovered in mammals including the PLC*β*, *γ*, *δ*, *ε*, *η*, and *ζ*. Each class of PLC is composed of many isotypes with distinct functions, domains, and regulatory mechanisms [44]. Based on the structure, they are further subdivided into 13 isoforms including PLC*β*1–4, *γ*1-2, *δ*1, *δ*3-4, *ε*, *ζ*, and *η*1–2 [31]. The structures of these PLC isoforms show conserved domains such as the X and Y domains that are responsible for catalytic activity, as well as regulatory specific domains including the PH domain, the C2 domain, and EF hand motifs involved in various biological functions of PLC isoenzymes [44, 45]. PLC isoforms are distinct in their activation mode, expression levels, cellular localization, and tissue distribution linking to a specific function for each isoform.

The spectrum of the expression of PLC isoforms in macrophages is phenotype-specific. It has been reported that in the case of human macrophages (derived from peripheral blood mononuclear cells), PLC*β*1–4, *γ*1-2, *δ*1, and *η*1-2 are expressed in unstimulated macrophages, PLC*β*1–3, *γ*1-2, *δ*1 and 3, and *η*1-2 are expressed in M1 macrophages, and PLC*β*1–3, *γ*1-2, *δ*3, and *η*1-2 are expressed in M2 macrophages. In addition, these PLC isoforms showed different subcellular localization in differently polarized macrophages [46]. The distinct expression spectrum and subcellular localization of these PLC isoforms reflect the diverse roles that they play in the regulation of the inflammatory response.

## 3. The Role of PLC*β* in Macrophage-Mediated Inflammatory Response

Macrophages express all the four PLC*β* isoforms orchestrating the Ca^2+^ signaling [47, 48], for example, the clostridium difficile ToxB-stimulated Ca^2+^ signaling in macrophages is enhanced via PLC*β*-4 signaling, but depressed by the PLC*β*-3 signaling [49]. Ca^2+^ and Erk1/2 signaling play important roles in the regulation of inflammatory response. PLC*β* is involved in the activation of Erk1/2 signaling in macrophages. It has been demonstrated that the glyceryl ester of prostaglandins activates Erk1/2 signaling in a dose-dependent manner through a pathway that requires PLC*β* signaling [50].

Cell adhesion is required for monocyte differentiation into macrophages. In human cytomegalovirus- (HCMV-) infected monocytic THP-1 cells, the viral protein US28 promotes adhesion to the endothelial cells via the activation of PLC*β*/PKC signaling cascade. Therefore, it is possible that PLC*β* signaling may promote the differentiation of monocytes to macrophages via cell adhesion [51]. U73122 is a pan inhibitor for PLC isoforms. We have demonstrated that U73122 inhibits PMA-induced human promonocytic U937 cell adhesion, as well as the differentiation into macrophages [29]. These two independent studies indicated that PLC signaling regulates cell adhesion and the differentiation of monocytes to macrophages.

It has been reported that LPS suppresses PLC*β*-2 and *β*-1 expression in macrophages in an MyD88-dependent manner, and the suppressed PLC*β*-2 plays an important role in switching M1 macrophages into an M2-like state [52, 53], suggesting that PLC*β*-2 signaling is closely involved in macrophage polarization.

PLC*β* signaling broadly regulates the expression of proinflammatory cytokines or chemokines in diverse cell cultures. The binding of HIV-1 envelope glycoprotein gp120 to CCR5 leads to PLC*β*-1 nuclear localization which promotes the release of chemokine CCL2 by macrophages [54], suggesting that activation of PLC*β*-1 signaling stimulates the expression of CCL2 in macrophages. PLC*β*-3 regulates IL-8 expression in bronchial epithelial cells via TLR-mediated activation of calcium signaling and NF-*κ*B pathway [55]. However, whether PLC*β*-3 regulates cytokine expression in macrophages has not been reported.

In summary, in macrophages, PLC*β*-1 signaling regulates the expression of CCL2, and PLC*β*-2 signaling regulates cell polarization, while PLC*β*-3 and PLC*β*-4 signaling regulates Ca^2+^ signaling with opposite effect.

## 4. The Involvement of PLC*γ* in Macrophage-Mediated Inflammatory Response

There are two main isoforms of PLC*γ* expressed in humans, PLC*γ*-1 and PLC*γ*-2, which regulate the development and functions of various hematopoietic cells [56, 57], for example, PLC*γ*1 regulates T cell activation and development through interaction with T cell receptor (TCR), and PLC*γ*-2 regulates development and maturation of B cells via interaction with pre-B cell receptor (BCR), reviewed by Nakamura and Fukami [31]. PLC*γ*-1 and PLC*γ*-2 are activated downstream of receptor (RTK) and nonreceptor tyrosine kinases, with tyrosine phosphorylation of PLC*γ* as the major mechanism. However, there is a novel mechanism towards the activation of PLC*γ*-2, which depends not on protein tyrosine phosphorylation, but on Rac GTPases [57–59]. Ubiquitously expressed PLC*γ*-1 is mainly activated by growth factors, including platelet-derived growth factor (PDGF), vascular endothelial growth factor (VEGF), epidermal growth factor (EGF), and fibroblast growth factor (FGF) [60]. PLC*γ*-1 binds to the tyrosine-phosphorylated receptors of EGF via its SH2 domain and downstream proteins via the SH3 domain [61]. We have recently identified that the exposure of macrophages to the proinflammatory cytokines TNF-*α* and IL-1*β*, as well as to influenza virus H1N1, leads to activation of PLC*γ*-1 in macrophages, which expands the spectrum of upstream stimulators for PLC*γ*-1 signaling [30]. Influenza virus H1N1 infection activates PLC*γ*-1 signaling through EGR receptor (EGFR) in alveolar epithelial cell line (A549 cells) [62]. But whether EGFR or the other RTKs act as an upstream activator for PLC signaling in macrophages is largely unknown. PLC*γ*-2, being predominantly expressed in hematopoietic cells, is activated by immune cell (T cell, B cell, and Fc) receptors associated with multiprotein complexes [60]. So PLC*γ*-1 and PLC*γ*-2 may be differentially activated to perform diverse functions.

Upon stimulation by LPS, TLR4 signaling induces proinflammatory cytokine production. Generally, TLRs regulate TLR-specific gene expression through the recruitment of distinct combinations of TLR/IL1R (TIR) domain-containing adaptor proteins, such as myeloid differentiation primary response gene 88 (MyD88), Toll/IL-1 receptor domain-containing adaptor protein (TIRAP), TIR domain-containing adaptor inducing IFN-*β* (TRIF), TRIF-related adaptor molecule (TRAM), and sterile *α*- and armadillo motif-containing protein (SARM) to form a signalosome, which activates downstream signals [63]. TLR4 is unique among these TLRs in its ability to utilize all of the TIR domain-containing adaptors and mediate activation of both MyD88-dependent and MyD88-independent (TRAM–TRIF-dependent) pathways [64–66], which are required to stimulate proinflammatory cytokine production in macrophages. In MyD88-dependent pathway, both MyD88 and TIRAP are required to activate NF-*κ*B and MAPK cascades and proinflammatory cytokine production [67, 68]. The MyD88-independent signaling events are controlled by TRIF and TRAM and induce IRF3-dependent type I interferon production [65, 69]. So in TLR4-mediated signaling, distinct adaptors are recruited to form diverse complexes which activate various downstream inflammatory signaling.

The involvement of PLC*γ* signaling in TLR4-mediated inflammation has been well identified. Currently, it is clear that PI(4,5)P2 plays an important role in TLR4 signaling. Mechanistically, TIRAP localizes to the plasma membrane by binding to PI(4,5)P2; there it recruits TLR4 and MyD88 to PI(4,5)P2-rich sites on the plasma membrane to form the TLR4 signalosome [69]. The distinct cellular localization of TLR4 complex leads to optional activation of MyD88-dependent or MyD88-independent signaling. Once TLR4 complex resides at the plasma membrane, the MyD88-dependent NF-*κ*B signaling is activated. Subsequently, the TLR-4 is delivered to the endosome compartment where MyD88-independent IRF3 signaling is activated [70]. The critical role that PI(4,5)P2 plays in TLR4 signaling is in linking TLR4 to PLC*γ* which controls the metabolism of PI(4,5)P2 [71]. Mechanisms for the regulation of LPS-induced TLR4 endocytosis and IRF3 activation by PLC*γ*-2 have been established: IP3, the cleavage product of PI(4,5)P2 by PLC*γ*-2, binding to IP3 receptors (IP3Rs) in the endoplasmic reticulum results in the release of Ca^2+^. The increased cytosolic Ca^2+^ is required for translocation of TLR4 from the plasma membrane to endosomes, where TRIF-dependent IRF3 activation takes place. In contrast, LPS-induced activation of NF-*κ*B pathway did not require PLC*γ*2-IP3-Ca^2+^ cascade [71]. Thus, signaling that affects TLR4 endocytosis could regulate TRIF-dependent signaling from endosome.

The LPS-binding protein CD14, together with TLR4 and MD-2, forms a multireceptor complex on the cell membrane [72]. CD14 controls the LPS-induced endocytosis of TLR4. LPS-induced clustering of CD14 triggers PI(4,5)P2 generation in macrophages [73], which may result in the activation of PLC*γ*2-IP3-Ca^2+^ cascade. The increase in cytosolic Ca^2+^, released from intracellular calcium stores, promotes the translocation of TLR4 from the plasma membrane to endosomes and so results in the activation of downstream inflammatory signaling. In addition, the CD14-dependent endocytosis pathway is regulated by several cytosolic regulators. Among them, the tyrosine kinase Syk and its downstream effector PLC*γ*-2 have been identified. The stimulation of Syk/PLC*γ*-2 signaling by CD14 triggers an influx of Ca^2+^ from the extracellular environment, which promotes internalization of TLR4 [72, 74]. So the endocytosis of TLR4 in response to CD14 clustering is partially regulated by the increased concentration of cytosolic Ca^2+^ originating either from intracellular calcium stores or the extracellular environment, which emphasizes the important role of Ca^2+^ in TLR4-mediated inflammation. In addition, these results support the idea that PLC*γ*-2 regulates the inflammatory response by controlling the cytosolic level of Ca^2+^. Apart from Ca^2+^, PKC signaling is also involved in TLR4 signaling in macrophages. It has been reported that the infection of both *P. aeruginosa* and *K. pneumoniae* activates TLR4/PLC*γ* cascades which in turn activates the PKC*α*/Jun N-terminal protein kinase (JNK)/NF-*κ*B axis and eventually induces the production of proinflammatory cytokines [75].

The generation of intracellular ROS in macrophages plays an important role in inflammation pertinent signaling transduction. The minimally oxidized LDL (mmLDL) stimulates ROS generation in macrophages through activation of NADPH oxidase 2 (Nox2), which is a suggested pathogenic mechanism for the development of atherosclerosis. It has been evidenced that mmLDL induces generation of ROS in macrophages through sequential activation of TLR4/Syk/PLC*γ*-1/PKC*α*/Nox2 cascade and thereby stimulates expression of proinflammatory cytokines IL-1*β*, IL-6, and RANTES [76, 77]. These studies indicate that PLC*γ*-1 regulates inflammatory response by the activation of PKC*α*, which is different from the role of PLC*γ*-2-dependent regulation of cytosolic Ca^2+^. Interestingly, we have recently shown that influenza virus H1N1 infection activates PLC*γ*-1 signaling and triggers ROS expression in human macrophages dU937 cells, which can be blocked by the PLC inhibitor U73122 [30]. Taken together, these two independent results reveal that PLC*γ* signaling regulates the generation of an important messenger ROS.

Phagocytosis by macrophages is a process that involves engulfment and clearing of invading microbial pathogens, concomitantly stimulating an inflammatory response leading to upregulation of inflammatory genes, such as TNF-*α*, IL-1*β*, and IL-12. The mechanism for Fc*γ*R-mediated phagocytosis has been extensively investigated. The ingestion of IgG-opsonized targets is initiated by the engagement and clustering of Fc*γ*Rs, which induce receptor tyrosine phosphorylation and subsequent activation of multiple downstream signaling pathways to promote the development of the phagocytic cup and the extension of pseudopods. The sequential process including cup formation, phagosome internalization, and phagolysosome formation is critical steps in the process of phagocytosis [78]. The translocation of PKC*ε* to phagosome is a critical step to regulate the rate of Fc*γ*R-dependent phagocytosis [79]. Diverse mechanisms regarding as to how Fc*γ*R-dependent phagocytosis is regulated by PLC*γ* signaling have been revealed, for example, PLC*γ*-1 is consistently concentrated at phagosomes and provides DAG to facilitate PKC*ε* localization to the phagosome [80]; Syk-dependent as well as Bruton's tyrosine kinase- (Btk-) and Tec-dependent activation of PLC*γ*-2 affects early and later stages of phagocytosis, respectively [78].

Peptidoglycan (PGN), the major cell wall component of Gram-positive bacteria, is able to stimulate proinflammatory cytokine production in macrophages. Normal human plasma from uninfected people contains low titer of anti-PGN IgG [81]. The anti-PGN IgG and Fc*γ*Rs are the key mediators of systemic inflammation in Gram-positive bacteria-induced sepsis [81, 82]. The binding of PGN to anti-PGN IgG triggers Fc*γ*R-mediated phagocytosis, which consequently leads to an inflammatory response [81]. In this mechanism, the phagocytosis of PGN-IgG-Fc*γ*R complex in macrophages is triggered by Ca^2+^ release from intracellular Ca^2+^ stores controlled by PLC*γ*-2 signaling [82, 83], suggesting that the regulation of intracellular calcium signaling by PLC*γ*-2 is involved in IgG-Fc*γ*R-mediated phagocytosis and cytokine production.

## 5. PLC*δ* Controls Phagocytosis

The PLC*δ*1-PH domain negatively regulates Fc*γ*RII-mediated cell spreading and phagocytosis through destabilizing PI(4,5)P2 availability in macrophages [84]. In addition, it has been reported that LPS stimulation reduces PLC*δ*1 expression at both mRNA and protein levels, an effect which would allow upregulation of the TLR4-induced proinflammatory cytokine production and Fc*γ*R-mediated phagocytosis [85]. These studies suggest that PLC*δ*1 negatively regulates TLR4/Fc*γ*R-mediated inflammatory response in macrophages. The roles of the other PKC*δ* isoforms including PKC*δ*3 and PKC*δ*4 in macrophage-mediated inflammation are not yet defined.

## 6. The Involvement of PLC*ε* in Inflammatory Response Has Been Characterized In Vivo, but Not in Macrophages

PLC*ε* is involved in a variety of signaling pathways and controls different cellular functions. Its role in carcinogenesis has been documented. With a PLC*ε* knockout mice model (PLC*ε*−/−), PLC*ε* has been identified as a novel tumor suppressor [86]. Also with this mouse model, it has been revealed that the airway inflammation induced by cigarette smoke *in vivo* was partially mediated by PLC*ε* signaling [87]. The PLC*ε* has also been convincingly demonstrated to regulate Ca^2+^ signaling in *β* cells and cardiomyocytes [88]. However, whether PLC*ε* is expressed in macrophages, as well as it is having any role in the macrophage-mediated inflammatory response, has not been identified.

## 7. Conclusions and Perspectives

Evidence accumulating from multiple studies has indicated that the PLC enzymes which functionally rely on the hydrolysis of PI(4,5)P2 to produce IP3 and DAG with subsequent modulation of calcium and PKC signaling regulate macrophage-mediated inflammatory response. The macrophage inflammatory response, such as the expression of inflammation-related genes and endocytosis, is controlled by calcium and/or PKC signaling. The PKC family contains ten isoforms with individual regulatory mechanism (summarized in Figure 1). Intracellular Ca^2+^ levels regulate multiple signaling pathways. In addition, the PLC family contains at least 13 members with specific activity for each one. Diversity of PKC family and the versatile Ca^2+^ signaling networks confers PLC enzyme multiple functions in the regulation of inflammatory response. Therefore, PLC enzymes are promising targets for the development of novel anti-inflammatory drugs.

**Figure 2:**
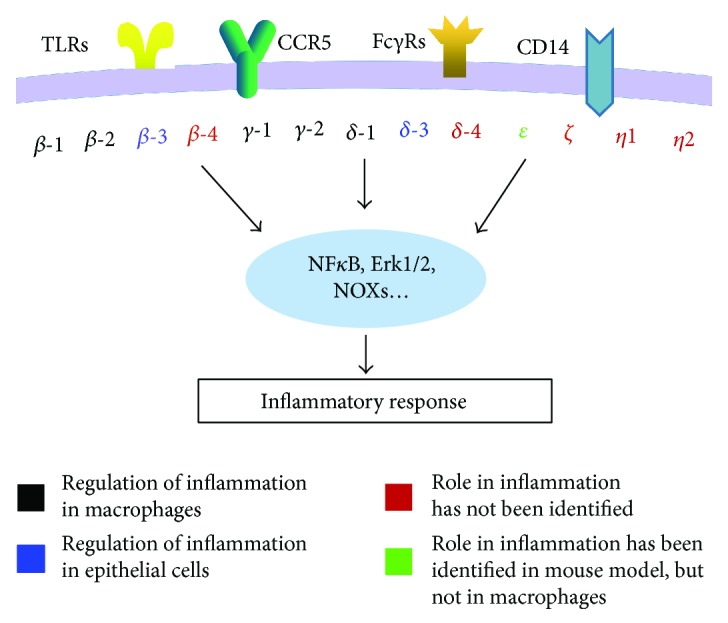
Schematic of macrophage-mediated inflammatory response through PLC signaling. PLC*β*1-2, PLC*γ*1-2, and PLC*δ* shown in black indicated that these PLC isoforms are expressed in macrophages and are involved in macrophage-mediated inflammatory response. PLC*β*3 and PLC*δ*3 shown in blue indicated that their involvement in inflammatory response has been identified in epithelial cell but not in macrophages. PLC*β*4, PLC*δ*4, PLC*ζ*, and PLC*η*1-2 shown in red indicated that whether they are involved in inflammatory response has not been identified. PLC*ε* shown in green indicated that the involvement of inflammatory response has been identified with mouse model, in vivo. But whether it regulates inflammatory response in macrophages has not been identified.

Macrophages express various receptors, such as TLRs, CD14, and Fc*γ*Rs, which have been identified as important upstream activators of PLC signaling (summarized in Figure 2). These receptors, such as CD14 and TRL4, may independently or collaboratively regulate the same or distinct PLC isoforms. In addition, some PLC isoforms may have opposite or synergistic effects on the same downstream signaling, for example, the concentration of intracellular Ca^2+^ is increased by PLC*β*-4 signaling, but decreased by PLC*β*-3. These studies indicate the complexity of the PLC-dependent signaling in the inflammatory response, and further research on PLC-dependent functions will contribute towards our understanding of the underlying mechanism of some inflammatory diseases.
